# Clonality analysis suggests that early-onset acute lymphoblastic leukaemia is of single-cell origin and implies no major role for germ cell mutations in parents

**DOI:** 10.1038/sj.bjc.6690440

**Published:** 1999-05

**Authors:** F Rinaldi, R J Mairs, T E Wheldon, F Katz, J M Chessells, B E Gibson

**Affiliations:** Department of Radiation Oncology, University of Glasgow, CRC Beatson Laboratories, Garscube Estate, Bearsden, Glasgow, G61 1BD, UK; Departments of Child Health and Haematology and Oncology, Royal Hospital for Sick Children, Glasgow, Yorkhill, G3 8SJ, UK; Department of Clinical Physics, University of Glasgow and West Glasgow Hospitals University NHS Trust, Western Infirmary, Glasgow, G11 6NT, UK; Department of Haematology and Oncology, The Hospital for Sick Children, Great Ormond Street, London, WC1, UK

**Keywords:** clonality, acute lymphoblastic leukaemia, germ cell mutations

## Abstract

Childhood leukaemia presenting at a young age has been suspected of resulting from a leukaemogenic mutation in parental germ cells, either spontaneously or due to the exposure of a parent to leukaemogenic environmental hazards, particularly ionizing radiation. Mathematical modelling of leukaemogenesis suggests that any such patient would be especially prone to multiple independent leukaemogenic events leading to multiclonality in terms of cell of origin (analogous to bilaterality in familial retinoblastoma). To test this hypothesis we have carried out a search for multiclonal leukaemogenesis in infant and childhood acute lymphoblastic leukaemia (ALL). We used a polymerase chain reaction-based analysis of the X-linked monoamine oxidase A (MAOA) gene locus to study the clonality of marrow samples obtained from female paediatric ALL patients at the time of disease presentation. We obtained presentation samples from 102 patients of whom 72 were found to be informative at the MAOA locus. These included 20 infant leukaemias (< 1 year at diagnosis). Sixty-six samples were found to be unequivocally monoclonal while the remaining six could not, with certainty, be assigned a clonal origin. We also obtained bone marrow aspirates at first relapse as well as at presentation from eight patients. In each case the same pattern of X-linked allelic inactivation was observed at both time points of the course of the disease. No evidence was found for leukaemic multiclonality in any age group at presentation or for leukaemic ‘clone-switching’ in relapse. These findings suggest that both infant and childhood ALL is of single-cell origin and implies that leukaemic predisposition resulting from germ cell mutation is unlikely to have a major role in their pathogenesis. © 1999 Cancer Research Campaign


*British Journal of Cancer* (1999) **80**(5/6), 909–913
© 1999 Cancer Research Campaign
Article no. bjoc.1998.0440

### Clonality analysis suggests that early-onset acute lymphoblastic leukaemia is of single-cell origin and implies no major role for germ cell mutations in parents

**F Rinaldi[1], RJ Mairs[1,2], TE Wheldon[1,4], F Katz[5], JM Chessells[5] and BE Gibson[3]**

[1]Department of Radiation Oncology, University of Glasgow, CRC Beatson Laboratories, Garscube Estate, Switchback Road, Bearsden, Glasgow G61 1BD, UK; Departments of [2]Child Health and [3]Haematology and Oncology, Royal Hospital for Sick Children, Glasgow, Yorkhill, Glasgow G3 8SJ, UK; [4]Department of Clinical Physics, University of Glasgow and West Glasgow Hospitals University NHS Trust, Western Infirmary, Glasgow G11 6NT, UK; [5]Department of Haematology and Oncology, The Hospital for Sick Children, Great Ormond Street, London WC1, UK

**Summary** Childhood leukaemia presenting at a young age has been suspected of resulting from a leukaemogenic mutation in parental germ cells, either spontaneously or due to the exposure of a parent to leukaemogenic environmental hazards, particularly ionizing radiation. Mathematical modelling of leukaemogenesis suggests that any such patient would be especially prone to multiple independent leukaemogenic events leading to multiclonality in terms of cell of origin (analogous to bilaterality in familial retinoblastoma). To test this hypothesis we have carried out a search for multiclonal leukaemogenesis in infant and childhood acute lymphoblastic leukaemia (ALL). We used a polymerase chain reaction-based analysis of the X-linked monoamine oxidase A (MAOA) gene locus to study the clonality of marrow samples obtained from female paediatric ALL patients at the time of disease presentation. We obtained presentation samples from 102 patients of whom 72 were found to be informative at the MAOA locus. These included 20 infant leukaemias (< 1 year at diagnosis). Sixty-six samples were found to be unequivocally monoclonal while the remaining six could not, with certainty, be assigned a clonal origin. We also obtained bone marrow aspirates at first relapse as well as at presentation from eight patients. In each case the same pattern of X-linked allelic inactivation was observed at both time points of the course of the disease. No evidence was found for leukaemic multiclonality in any age group at presentation or for leukaemic 'clone-switching' in relapse. These findings suggest that both infant and childhood ALL is of single-cell origin and implies that leukaemic predisposition resulting from germ cell mutation is unlikely to have a major role in their pathogenesis.

**Keywords:** clonality; acute lymphoblastic leukaemia; germ cell mutations

Germ line mutations provide a recognized oncogenic pathway for some paediatric solid tumours, notably retinoblastoma and nephroblastoma (Narod et al, 1991). Tumours resulting from germ line mutations have characteristic young age of onset and propensity to multiclonal genesis (e.g. bilaterality in retinoblastoma) whether or not familial associations are apparent. Interest in the possible role of leukaemogenic germ cell mutations in childhood leukaemia was stimulated by the 'Gardner hypothesis' (Gardner et at, 1990) postulating increased leukaemia risk in the offspring of fathers receiving gonadal irradiation whilst working at the Sellafield nuclear establishment, although this hypothesis is not supported by additional epidemiological evidence now available (Kinlen, 1993; Little et at, 1995). More recently, Shu et al (1994) reported a statistical association between infant leukaemia and preconceptual exposure of fathers to diagnostic X-rays, and postulated a role for radiation-induced germ cell mutations. The possibility of 'paternal preconception irradiation' (PPI) having a causal role in leukaemia was reviewed by the Committee on the Medical Aspects of Radiation in the Environment (COMARE) who concluded that 'there is no doubt that the hypothesis of PPI causing cancer in offspring can be sustained in principle' although this did not seem to be the explanation for the increased incidence of childhood leukaemia in the vicinity of the Sellafield nuclear Plant (COMARE, 1996). The existence of vertically transmissible leukaemia-predisposing mutant genes has been demonstrated in transgenic mice (Langdon et al, 1986; Heisterkamp et al, 1990; Griffiths et al, 1992) and it has been shown that these may give rise to independently transformed leukaemic clones (Langdon et al, 1986). In very recent experimental studies, Lord et al (1998) have reported that contamination of male mice with the α-emitter $^{239}$Pu results in an increased susceptibility to leukaemia induction by methyl-nitroso-urea in first-generation offspring.

It seems possible that germ cell mutations induced by environmental factors (e.g. natural background radiation or diagnostic exposures), perhaps followed by subsequent insults, could similarly contribute to the pathogenesis of leukaemias and other neoplasms in human populations. Acute lymphoblastic leukaemia (ALL) is rarely familial but ALL in infants (< 12 months) or very young children (< 24 months) may be suspected as genetically predisposed because of age of onset. Infant leukaemias have distinctive properties: prognosis is poor for ALL (Chessels et al, 1994) and characteristic aberrations at chromosome 11q23 are often observed, usually involving rearrangements of the MLL gene (Piu et al, 1995; Greaves, 1996). Such rearrangements are not heritable and have been shown to originate in utero (Ford et al, 1993; Mahmoud et al, 1995), but this does not exclude the

*Received 17 September 1998*
*Revised 7 December 1998*
*Accepted 9 December 1998*

*Correspondence to*: RJ Mairs, Department of Radiation Oncology, University of Glasgow, CRC Beatson Laboratories, Garscube Estate, Switchback Road, Bearsden, Glasgow G61 1BD, UK

inheritance of a preceding and possibly predisposing mutation. Very recently, Gale et al (1997) have demonstrated by polymerase chain reaction (PCR) methodology the presence at birth of small numbers of leukaemic cells in neonatal ‘blood spots’ of infants who subsequently developed clinically overt leukaemia. This finding demonstrates the prenatal origin of these leukaemias but does not distinguish preconceptual predisposing events from events occurring exclusively in utero.

Here we report clonality studies which are relevant to the role of germ line mutations in the genesis of ALL. The rationale of the study was to seek evidence of multiclonal origin of early-onset ALL analogous to bilateral presentations of retinoblastoma (Rb) in which one Rb gene is mutated in the germ line, leaving the other allele to be mutated somatically (Knudson, 1971; Narod et al, 1991). ALL is also hypothesized to result from two independent mutations (Greaves and Chan, 1986) although the genetic targets are yet to be identified. It is of course possible that this is an underestimate of mutation number, but it is plausible that only a small number of mutations would be required for a neoplasm which presents in early childhood. We have mathematically modelled ‘two hit’ leukaemogenesis with both mutations occurring somatically, or with one transmissible via the germ line. With parameters chosen to simulate the observed age-incidence of ALL, these studies predict that inheritance of the first mutation would cause multiple independent leukaemogenic transformation in the haematopoietic cells of each individual, typically resulting in ten or more independently arising leukaemic clones by time of diagnosis (Wheldon et al, 1997). Multiclonality following a predisposing germ cell mutation is easily understood as resulting from the large number of somatic cells which need only acquire one further mutation to experience malignant transformation. The predicted number of leukaemic clones (> 10) is greater than the number of independently occurring tumours (about 3) estimated for familial retinoblastoma (Knudson, 1971) because the higher incidence of ALL requires higher mutation rates to be assigned to the two-mutation model (Wheldon et al, 1997). As in Knudson’s model, a small proportion of genetically predisposed leukaemias may present (by chance) as a single clone: however, the present modelling studies (Wheldon et al, 1997) suggest that the fraction of heritably predisposed ALL patients who would present in this way is close to zero. Therefore, we expect multiclonality in almost all presenting ALL cases who have inherited a first mutation (provided of course that the two-mutation model is valid). A clinical corollary of this model is that early-onset leukaemias may be difficult to treat effectively because of repeated emergence of new leukaemic clones.

The investigation reported here provides a test of the multiclonality hypothesis and indirectly of the hypothesis that early-onset leukaemias result from the inheritance of one of two possible mutations.

### CLONALITY ANALYSIS BY X-CHROMOSOME INACTIVATION

We tested the multiclonality hypothesis experimentally using X-chromosome inactivation analysis. This is based upon the polymorphic differentiation between maternal and paternal X-chromosomes and the discrimination between active and inactive X-chromosomes, by differential methylation. The first such studies, by Fialkow (1976), utilized the expression of a rare polymorphism at the X-linked, glucose-6-phosphate dehydrogenase gene locus. To improve upon the low informative rate associated with this technique, Vogelstein et al (1987) employed Southern blot analysis of differentially methylated, X-chromosomal loci containing restriction fragment length polymorphisms. However, substantial amounts of intact DNA (5–20 mg) are required for such analyses and sufficient quantities are not readily available from pathological specimens. Therefore, the PCR has been applied to enhance the sensitivity of this approach. Informative X-chromosomal regions include sequences of the genes encoding phosphoglycerate kinase (PGK) (Gilliland et al, 1991), androgen receptor (AR) (Allen et al, 1992) and monoamine oxidase A (MAOA) (Hendriks et al, 1992). The associated heterozygosity rates are 32%, 90% and 75% respectively. Because limited amounts of leukaemic sample material were available, PCR methodology was used in the present study. We based our analysis on the MAOA locus because it has a higher rate of heterozygosity than the PGK locus and its reliability is not limited by incomplete digestion of active alleles: a shortcoming associated with AR-PCR (Mashal et al, 1993).

### MATERIALS AND METHODS

### Reagents

*Taq* polymerase, restriction enzymes and dNTPs were purchased from Promega (Southampton, UK). Other reagents, unless otherwise stated, were obtained from Sigma (Dorset, UK).

### DNA samples

A total of 102 samples of bone marrow (each containing less than 5 mg of DNA) from female childhood ALL patients were received from the Department of Haematology, Sick Children’s Hospital, Yorkhill, Glasgow; the Department of Haematology and Oncology, Institute of Child Health, London; and the Department of Medicine, Section of Hematology and Oncology, University of Chicago. DNA, purified from bone marrow aspirates of chronic myelocytic leukaemia patients, was acquired from Ms Anne Sproul, Haematology Department, Royal Infirmary, Glasgow. Blood and marrow were obtained originally from patients for diagnostic purposes. DNA was also prepared from the peripheral blood samples of 46 healthy, adult, female and five male volunteers.

High molecular-weight DNA was purified from the mononuclear cell fractions of marrow samples as described previously (Sambrook et al, 1989). Ethanol-precipitated DNA was rehydrated in sterile water and DNA concentration was determined by $E_{260}$ measurements and by fluorescence assay using the Hoeschst 33258 method (Cooper and Stratton, 1991).

### Determination of clonality

*MAOA-PCR*

Individuals whose clonality status is assessable by this method are heterozygous with respect to a GT-dinucleotide/variable number tandem repeat (VNTR) region of the MAOA gene (Hendriks et al, 1992). All DNA samples were screened by amplification at the MAOA locus as described below and those found to be informative were subjected to clonality determination.

The method relies upon the presence of several sites recognized by the methylation-sensitive restriction endonuclease *Hpa*II. These are located 5′ relative to a highly polymorphic region of the MAOA gene. Because inactive X-chromosomes are extensively

 *© Cancer Research Campaign 1999*

methylated at these sites, only the active X-chromosomes are cleaved by *Hpa*II. The undigested MAOA alleles are PCR-amplified using primers that flank the 5′ site of differential methylation and the 3′ hypervariable region, which is composed of a VNTR motif and a GT microsatellite. *Sac*I digestion produces fragments of 258–388 bp, depending on the number of VNTR and dinucleotide repeats. If *Hpa*II digestion is allowed to proceed to completion, PCR amplification and subsequent *Sac*I digestion produce one allelic band from monoclonally derived cell samples, whereas two bands of reduced intensity are generated from polyclonal cell populations.

Two reactions were prepared: (a) 100 ng samples of genomic DNA were digested with 5 units of *Hpa*II; (b) the same amount of DNA was incubated with enzyme digestion buffer in the absence of enzyme. All reactions were in a total volume of 10 µl and all incubations were carried out at 37°C for 18 h. *Hpa*II digestion was terminated by heat inactivation at 65°C for 5 min. All of the reaction mixture was included in the subsequent PCR amplification.

PCR conditions were a modification of those reported (Hendriks et al, 1992). Briefly, amplification was performed in a total volume of 100 µl containing the following components: 1.5 mM magnesium chloride, 50 mM potassium chloride, 10 mM Tris–HCl (pH 9.0) and 0.1% (v/v) Triton X-100. After initial denaturation at 94°C for 5 min, 35 amplification cycles were performed using a Hybaid Omnigene thermocycler (Teddington, UK). Each cycle comprised 1 min at 94°C, 1 min at 54°C and 2 min at 70°C. The following primers were used: 5′-CAATAAATGTCCTACACCTT-3′ and 5′-ACATTCTAAACCTAATAACTC-3′ (Oswell, Southampton, UK). After amplification, PCR products were extracted with phenol–chloroform, ethanol precipitated and redissolved in 10-µl digestion buffer containing 8 units of *Sac*I. Digestion was carried out for 2 h at 37°C, followed by heat inactivation at 65°C for 5 min. To facilitate allele detection, digests were electrophoresed through 4% (w/v) fine resolution Metaphor agarose gel (FMC Bioproducts, Kent, UK) and stained with ethidium bromide. The relative intensities of the bands were determined using photographic negatives of the stained gels. Densitometry was performed on a Molecular Dynamics computing densitometer (Sevenoaks, Kent, UK) and images were analysed using the Quantity 1P Programme (Pharmacia Herts, UK).

### *Southern hybridization*

Clonality assessment, by the analysis of differential methylation at the 5′ region of the PGK locus using the *Bst*X1 polymorphism, was performed as described (Vogelstein et al, 1987). Briefly, 6.25 µg of genomic DNA were digested with *Pst*I and *Bst*XI. After precipitation and redissolution, half of the DNA was digested with *Hpa*II and half was left undigested. Fragments were resolved in 1.0% (w/v) agarose gels and blotted on to Hybond N membranes (Amersham International plc, Bucks, UK) before hybridization to a PGK probe. This was an 800-bp *Bam*HI/*Eco*RI fragment from the 5′ end of the PGK gene, labelled to a specific activity of $5 \times 10^8$ cpm $\mu g^{-1}$.

### RESULTS

To ensure a true assignment of clonality, it was essential that *Hpa*II digestion of active MAOA alleles, prior to amplification, proceeded to completion. This was verified by the simultaneous processing of male DNA, whose single active X-allele was not detectable after *Hpa*II digestion.

The validity of the determination of the clonal status of ALL samples by MAOA-PCR was confirmed by comparison with clonal assessment using Southern blot analysis of the PGK locus. Three chronic myelocytic leukaemia DNA samples (monoclonal) and two normal peripheral blood DNA samples (polyclonal) were analysed by both methods. The results of the Southern blot analysis were, in every case, in agreement with those obtained by MAOA-PCR.

**Table 1** Age distribution and diagnosis of informative ALL patients

| | Age (years) | | |
|---|---|---|---|
| **Diagnosis** | **< 1** | **1–2** | **> 2–13 <** |
| cALL | 5 | 4 | 32 |
| Null ALL | 12 | 3 | 4 |
| Pre B-ALL | 2 | 0 | 2 |
| T-ALL | 1 | 0 | 1 |

To study the clonal origin of infant ALL, we attempted to PCR amplify at the MAOA locus, samples of mononuclear cellular DNA from bone marrow aspirates collected at the time of disease presentation. Of the samples from 102 female paediatric ALL patients (age range 1 month to 13 years), 72 were heterozygous at the MAOA polymorphic locus. This is equivalent to a heterozygosity rate of 71%. The age distribution of the different categories of ALL patients is shown in Table 1.

Using the MAOA-PCR-based clonality assessment, 66 out of 72 informative presentation ALL samples were found to be monoclonal. The upper allele was inactive in 29 and the lower allele was inactive in 37. The remaining six samples yielded ambiguous results: the allele intensity ratios in the absence of *Hpa*II digestion were approximately unity, whereas after *Hpa*II digestion, preferential but incomplete loss of one allele was observed resulting in allele intensity ratios greater than 7:1 or less than 1:5.

To determine whether alteration in clonal status occurred during the course of the disease, another means of assessing the origin of leukaemic clones was employed. This involved a comparison of the allele inactivation patterns of DNA isolates from presentation and relapse marrow. Thirteen such pairs of samples (patient ages < 2 years) were obtained. Of these, eight were informative. In every case, the inactive allele in presentation marrow was the same as the inactive allele observed in material obtained at the time of relapse.

### DISCUSSION AND CONCLUSIONS

To test the possible multiclonality of early onset ALL, we studied clonal origin by MAOA-PCR methodology. Assignment of clonality was possible in samples from 27 informative early-onset patients (0.1–2.0 years) and 39 informative, older children (2.1–12.0 years), whereas six samples gave results which could not be confidently evaluated. This is the largest study of clonality by X-chromosome inactivation so far reported for childhood ALL. All of the evaluable patient samples in both age groups were found to be monoclonal. This demonstration of single-cell origin for childhood ALL is in agreement with the findings of an earlier study of eight female children, aged 2–13 years (Dow et al, 1985). The latter determination of clonality was based upon the analysis of X-chromosome-encoded, glucose-6-phosphate dehydrogenase isoenzymes.

A possible difficulty with the interpretation of clonality analysis in leukaemia is that the disease does not present with individually distinct tumours, and a leukaemic cell population which contains

© Cancer Research Campaign 1999

several distinct clones may be numerically dominated at presentation by a single clone – the earliest to arise, or possibly the most rapidly growing – giving a spurious finding of monoclonality, if the co-existing clones fall below the detection threshold of the assay. It is difficult to exclude this eventuality on the basis of a single sample for each patient. However, we had available to us 66 evaluable patients, including 20 infants, and it would be surprising that we were unable to detect clonal co-existence in any of them if true multiclonality were in fact a common occurrence.

An alternative strategy for detection of multiclonal leukaemogenesis requires the provision of sequential marrow samples from the same patient, i.e. at first presentation and at subsequent relapse. This provides the opportunity for 'clone-switching' in a multiclonal setting – the clone present at relapse being different from that occurring at first diagnosis, either because the selection of a different (pre-existing) clone has been favoured by therapy or because a new independent clone has arisen subsequent to therapeutic eradication of the initial clone. We obtained presentation and relapse marrow samples from 13 patients < 2 years in this series, eight of whom proved to be informative. In all eight cases, the clone present at relapse did not differ in relation to X-inactivation from the original clone, providing no evidence of multiclonality. It is not possible to calculate precisely the probability of this occurring by chance, because we have no way to quantify the probability that a co-existing minor clone would be resistant to therapy, or that the original dominant clone had been eliminated. Suppose, however, that the original clone were indeed eliminated, and that relapse is due to a newly arising clone (on our mathematical model this is quite a common event). If the newly arising clone had an equal chance of either X-chromosome's being inactivated, then the probability of observing the same pattern of X-inactivation at presentation and relapse in each of eight patients is $0.5^7$, or 0.008. Despite the restrictive assumptions of the calculation, it seems unlikely that we would have failed to observe 'clone-switching' in any of eight cases, if multiclonal leukaemogenesis were usually occurring. A further area of uncertainty concerns the mathematical model which predicts multiclonality were a germ cell mutation to be inherited (Wheldon et al, 1997). We have previously mentioned that more than two leukaemogenic mutations may in fact be required, although this does not seem plausible in the case of infant leukaemia. We should also note that our model does not allow for the inheritance of 'modifier genes' which alter the mutability of other genes – currently described as 'caretaker genes' by Kinzler and Vogelstein (1997). Inheritance of mutant caretaker genes could provide a general tendency to genetic instability in offspring, without necessarily resulting in multiclonality, but it is also unlikely that the effects of such genes would be confined to leukaemogenesis.

Because of the reservations above, we are not entitled to conclude that our findings categorically exclude the possibility of multiclonal leukaemogenesis, or the role of germ cell injury. However, we have failed to find evidence of multiclonal leukaemogenesis in the largest study of clonality in childhood ALL to date, despite focusing on early-onset leukaemias, and seeking additional evidence of 'clone-switching' between presentation and relapse. It therefore seems unlikely (though not impossible) that germ cell mutations, from which multiclonal leukaemogenesis would have been expected, play a major role in the pathogenesis of even the youngest cases of ALL.

Since it is now clear from other studies (Ford et al, 1993; Mahmoud et al, 1995; Gale et al, 1997) that the critical leukaemogenic events in early-onset ALL occur prenatally, it seems probable that these events occur exclusively in-utero. (In-utero mutational events would not result in multiclonality unless affecting the early embryo prior to the development of yolk-sac haemopoesis.) Further investigation of leukaemogenic hazards to the embryo or fetus seems warranted.

### ACKNOWLEDGEMENTS

This work was supported by grant number 3100791 from the Coordinating Committee on Health Aspects of Radiation Research (CCHARR). Additional support was provided by the Cancer Research Campaign. We are grateful to Dr WM Crist (Section of Medicine, University of Chicago) for providing some of the bone marrow samples and to Prof. OB Eden (Manchester) for helpful advice and encouragement.

### REFERENCES

Allen RC, Zoghbi HY, Mosely AB, Rosenblatt HM and Belmont JW (1992) Methylation of Hpa II and Hha I sites near the polymorphic CAG repeat in the human androgen receptor gene correlates with X chromosome inactivation. *Am J Hum Genet* **51**: 1229–1239

Chessels JM, Eden OB, Bailley CC, Lilleyman JS and Richards SM (1994) Acute lymphoblastic leukaemia in infancy: experience in MRC UKALL trials. *Leukemia* **8**: 1275–1279

Committee on Medical Aspects of Radiation in the Environment (COMARE). (Chairman: Prof. BA Bridges) Fourth report. *The Incidence of Cancer and Leukaemia in Young People in the Vicinity of the Sellafield Site, West Cumbria*. Further studies and an update of the situation since the publication of the report of the Black Advisory Group in 1984. Department of Health: London

Cooper CS and Stratton MR (1991) Extraction and enzymatic amplification of DNA from paraffin-embedded specimens. In *Protocols in Human Molecular Genetics*, Mathew CG (ed), pp. 133–140. The Humana Press: Clifton, NJ

Dow LW, Martin P, Moohr J, Greenberg M, MacDougall LG, Najfeld V and Fialkow PJ (1985) Evidence for clonal development of childhood acute lymphoblastic-leukaemia. *Blood* **66**: 902–907

Fialkow PJ (1976) Clonal origin of human tumours. *Biochem Biophys Acta* **458**: 283–321

Ford AM, Ridge S, Cabrera ME, Mahmoud H, Steel CM, Chan LC and Greaves M (1993) In utero rearrangements in the trithorax-related oncogene in infant leukaemias. *Nature* **363**: 358–360

Gale KB, Ford AM, Repp R, Borkhardt A, Keller C, Eden OB and Greaves MF (1997) Backtracking leukaemia to birth: identification of clonotypic gene fusion sequences in neonatal blood spots. *Proc Natl Acad Sci USA* **94**: 13950–13954

Gardner MJ, Snee MP, Hall AJ, Powell CA, Downes S and Terrell JD (1990) Results of case control study of leukaemia and lymphoma among young people near Sellafield nuclear plant in West Cumbria. *Br Med J* **300**: 423–429

Gilliland DG, Blanchard KL, Levy J, Perrin S and Bunn HF (1991) Clonality in myleproliferative disorders: analysis by means of the polymerase chain reaction. *Proc Nat Acad Sci USA* **88**: 6848–6852

Greaves MF (1996) Infant leukaemia biology, aetiology and treatment. *Leukemia* **10**: 372–377

Greaves MF and Chan LC (1986) Is spontaneous mutation the major cause of childhood acute lymphoblastic leukaemia? *Br J Haematol* **64**: 1–13

Griffiths SD, Healy LE, Ford AM, Bennett CA, Voncken JW, Heisterkamp N, Groffen J and Greaves MF (1992) Clonal characteristics of acute lymphoblastic cells derived from *BCR/ABL*p190 transgenic mice. *Oncogene* **7**: 1391–1399

Heisterkamp N, Jenster G, Ten Hoeve J, Zovich D, Pattengale PK and Groffen J (1990) Acute leukaemia in *bcr/abl* transgenic mice. *Nature* **344**: 251–253

Hendriks RW, Chen Z-Y, Hinds H, Scuurman RKB and Craig IW (1992) An X chromosome inactivation assay based on the differential methylation of a CpG island coupled to a VNTR polymorphism at the 5′ end of the monoamine oxidase A gene. *Hum Mol Genet* **1**: 187–194

Kinlen LJ (1993) Can paternal preconceptional radiation account for the increase in leukaemia and non-Hodgkin's lymphoma in Seascale? *Br Med J* **306**: 1718–1721

Kinzler KW and Vogelstein B (1997) Cancer susceptibility genes: gatekeepers and caretakers. *Nature* **386**: 761–763

© Cancer Research Campaign 1999

Knudson AG (1971) Mutation and cancer: statistical study of retinoblastoma. *Proc Natl & Acad Sci USA* **68**: 820–823

Langdon WY, Harris AW, Cory S and Adams JM (1986) The c-myc oncogene perturbs lymphocyte-B development in E-mu-myc transgenic mice. *Cell* **1**: 11–18

Little MP, Charles MW and Wakeford R (1995) A review of the risks of leukaemia in relation to parental preconception exposure to radiation. *Health Phys* **68**: 299–310

Lord B, Woolford LB, Wang L, Stones VA, McDonald D, Lorimore SA, Papworth D, Wright EG and Scott D (1998) Tumour induction by methyl-nitroso-urea following preconceptional paternal contamination with plutonium-239. *Br J Cancer* **78**: 301–311

Mahmoud HH, Ridge SA, Behm FG, Pui C-H, Ford AM, Raimondi SC and Greaves MF (1995) Intrauterine monoclonal origin of neonatal concordant acute lymphoblastic leukemia in monozygotic twins. *Med Ped Onc* **24**: 77–81

Mashal RD, Lester SC and Sklar J (1993) Clonal analysis by study of X chromosome inactivation in formalin-fixed paraffin-embedded tissue. *Cancer Res* **53**: 4676–4679

Narod SA, Stiller C and Lenoir GM (1991) An estimate of the heritable fraction of childhood cancer. *Br J Cancer* **63**: 993–999

Pui CH, Kane JR and Crist WM (1995) Biology and treatment of infant leukaemias. *Leukemia* **9**: 762–769

Sambrook J, Fritsch EF and Maniatis T (1989) *Molecular Cloning: a Laboratory Manual*, pp. 9.17–9.19. Cold Spring Harbour Laboratory Press: New York

Shu XO, Reaman GH, Lampkin B, Sather HN, Pendergrass TW and Robison LL (1994) Association of paternal diagnostic X-ray exposure with risk of infant leukemia. *Cancer Epidemiol Biomarkers Prev* **3**: 645–653

Vogelstein B, Fearon ER, Hamilton SR, Preisinger AC, Willard HF, Michelson AM, Riggs AD and Orkin SH (1987) Clonal analysis using recombinant DNA probes from the X-chromosome. *Cancer Res* **47**: 4806–4813

Wheldon EG, Lindsay KA and Wheldon TE (1997) A two-stage model for childhood acute lymphoblastic leukaemia: application to hereditary and non-hereditary leukaemogenesis. *Math Biosci* **139**: 1–24

*© Cancer Research Campaign 1999*
